# Isolated Rheumatoid Nodules: A Diagnostic Dilemma

**DOI:** 10.1155/2015/352352

**Published:** 2015-02-23

**Authors:** Michael Gale, Erin Gilbert, David Blumenthal

**Affiliations:** ^1^Department of Medicine, Woodhull Medical Center, Brooklyn, NY 11206, USA; ^2^Department of Dermatology, Woodhull Medical Center, Brooklyn, NY 11206, USA

## Abstract

We present a 27-year-old male with multiple nonpainful soft tissue masses over several metacarpals, bilateral elbows, the left wrist, and both knees since the age of 4. Physical exam was significant for firm, nonmobile, nodular growths over the extensor surfaces of bilateral elbows and knees and on the 2nd and 5th metacarpal phalangeal joints. Laboratory studies revealed an unremarkable rheumatoid factor, negative ANA screening and normal joint radiographs. Differential diagnosis included subcutaneous granuloma annulare (SGA), seronegative rheumatoid nodule, and calcinosis cutis. Biopsy is the only method to distinguish benign rheumatoid nodules from SGA. This case illustrates the importance of biopsy in diagnosis, an awareness of the potential complications, and the need for good follow-up.

## 1. Presentation

**Figure 1:**
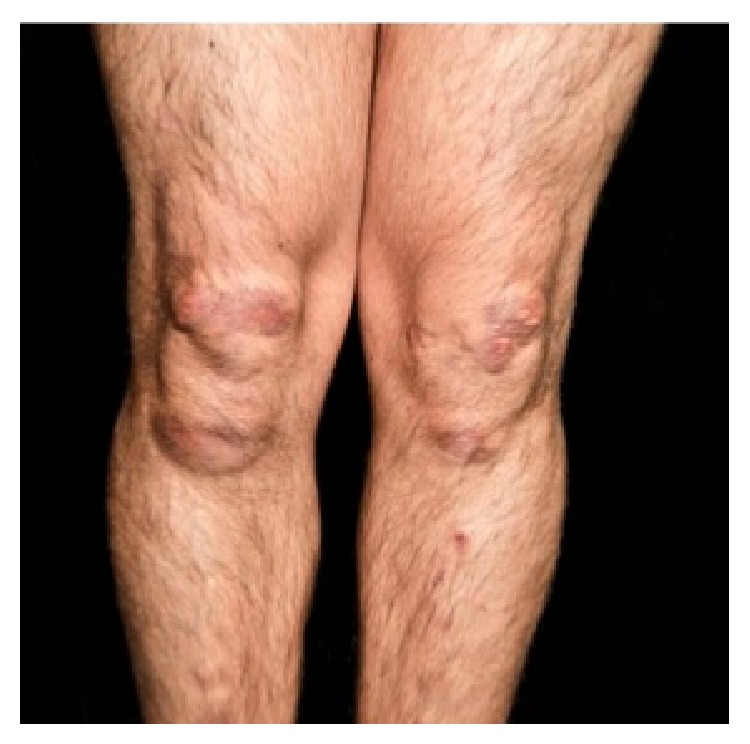
Bilateral lower extremities demonstrating multiple firm, nontender nodules over the knees and shins with overlying yellow plaques.

**Figure 2:**
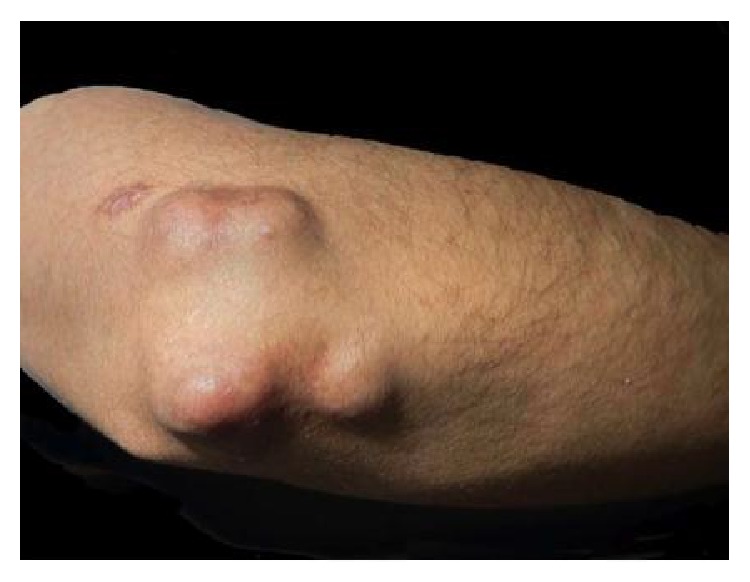
Right elbow with multiple firm nodules with overlying yellow-red papules.

**Figure 3:**
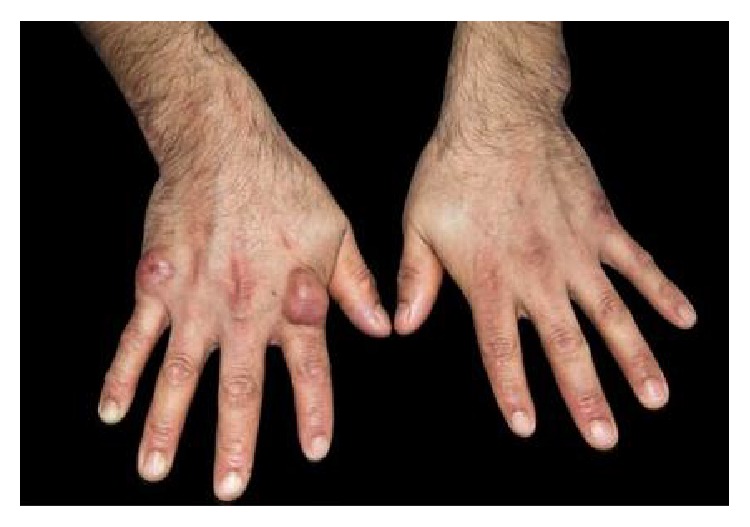
Bilateral hands with firm, nonmobile, nodular growths over the joints of the 2nd and 5th metacarpal phalangeal joints.

A 27-year-old male from Ecuador, with no significant past medical history presented to our clinic with multiple nonpainful soft tissue masses over several metacarpals, bilateral elbows, left wrist, and both knees (Figures 1, 2, and 3).

The patient stated that these lesions first appeared when he was four years old and had since grown in size and number. The nodules were nonpainful and nonpruritic and did not limit his movements. The patient wanted treatment for cosmetic reasons only. The patient denied alcohol, smoking, and illicit drug use. He worked in a kitchen after emigrating from Ecuador four years ago. He had no history of allergies and was not on any current medications. These nodules are consistent with a wide range of medical conditions ranging from autoimmune processes to infectious diseases. Consultation was requested from the rheumatology, dermatology and surgery departments.

## 2. Assessment

Physical exam was significant for firm, nonmobile, nodular growths over the joints on the extensor surfaces of the bilateral elbows and knees and on the 2nd and 5th metacarpal phalangeal joints. The masses were nontender and nonwarm, with nonspecific skin changes including multiple yellow nodules overlying the right elbow nodule (Figure 2).

The largest nodule was approximately 5 cm × 5 cm. There were also multiple deep seated nodules appreciated in both the right flank and inferior buttocks. The rest of the physical exam revealed no abnormalities except for persistent nystagmus. Laboratory studies revealed a negative ANA panel, negative Scl-70 AB, ESR 3 mm/h. The rheumatoid factor was 8 (normal value less than 13) and anti-CCP less than 16 units (normal value < 20). All other lab works were within normal range. The patient was HIV negative and hepatitis B negative with normal TSH and a unremarkable lipid panel. PTH was 34.39 pg/ml (normal value 15.0–65.0) and calcium 9.9 mg/dL. Radiologic images of bilateral hands, wrists, and elbows revealed no bony or articular abnormality. There were lobular soft tissue prominence over the proximal ulnas bilaterally, overlying the fifth metacarpal phalangeal joint on the right hand and overlying the fifth metacarpal phalangeal joint and distal ulna on the left hand (Figure 3). Joint space preservation was noted on all joints mentioned above without any abnormal calcification. It would have been desirable to obtain ultrasonography or MRI of the patients' hands and wrists to detect synovial inflammation or infraradiological erosions which can be present in the early diagnosis of rheumatoid arthritis. Unfortunately, the patient failed to follow up with any further radiologic imaging appointments.

## 3. Diagnosis

**Figure 4:**
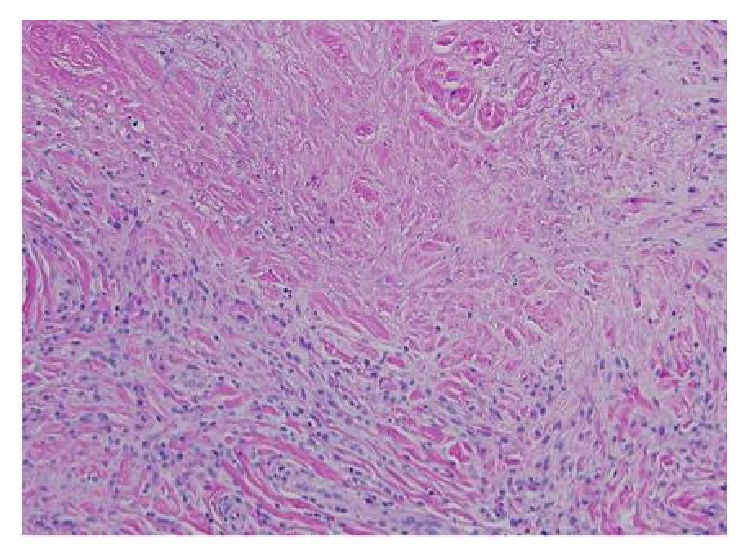
Histopathological evaluation of a nodule on the right hand (20x) shows a perivascular and interstitial infiltrate of lymphocytes and histiocytes surrounding areas of degenerated collagen and fibrin with mucin deposition.

It was initially suspected that our patient had rheumatoid nodules secondary to rheumatoid arthritis despite the lack of joint signs or symptoms on presentation. Although the laboratory workup revealed an unremarkable rheumatoid factor and negative ANA screening without radiographic disease, our differential diagnosis remained possible SGA, benign rheumatoid nodules, cat scratch, and calcinosis cutis. Our patient had an unusual presentation as the lesions on his lower extremities suggested that SGA was more likely than benign rheumatoid nodules. A punch biopsy was taken of one of the nodules. The initial report was inconclusive. However, it demonstrated a collagenized soft tissue mass composed of small and large geographic areas of collagen degeneration, surrounded by a histiocytic reaction. Bluish discolorations were noted, which indicated mucin accumulation. Pathological evaluation of a repeat biopsy noted that within the lower reticular dermis a perivascular and interstitial infiltrate of lymphocytes and histiocytes was seen. Histocytes surrounded areas of degenerated collagen and fibrin. A diagnosis of rheumatoid nodule was established (Figure 4).

The pathological appearance of rheumatoid nodules can appear almost identical in both the benign disease and in the setting of arthritis. Rarely rheumatoid nodules can be encountered in patients with no antecedent evidence of arthritis. Benign rheumatoid nodules are subcutaneous nodules morphologically and histologically identical to the ones appearing in patients with rheumatoid arthritis. They usually occur in healthy people without clinical, radiographic or serologic manifestations of any rheumatic illness [1]. Confirmation is established by a biopsy with wide excision of the nodule. A skin biopsy is the only method to distinguish benign rheumatoid nodules from SGA. The histology of the lesions of SGA may be indistinguishable from rheumatoid nodules [2]. Positive mucin staining is characteristic of SGA and a distinguishing test from rheumatoid nodules. This complicated the diagnosis in our case as initial biopsy indicated mucin accumulation. These lesions are benign, and although a relationship between SGA and rheumatoid arthritis is suggested, the current literature supports that SGA virtually never progresses to rheumatoid nodules. SGA is seen exclusively in children and lesions usually appear in the lower extremities, especially in the pretibial area, followed by the hands. The buttocks, forehead, and scalp are less commonly affected. Our case presented with lower extremity lesions; therefore on clinical presentation he appeared more likely to have SGA. Benign rheumatoid nodules are more usual in children than in adults and they are considered exceptional beyond the age of eighteen [3].

The diagnosis of benign rheumatoid nodules carries a good prognosis. Systemic disease is usually absent on follow-up of up to 20 years. However, on rare occasions patients seroconvert and become rheumatoid factor positive or even develop full systemic joint disease [4]. Berardinelli et al. [5] described 10 children with benign nodulosis in whom rheumatoid factor became positive between 2 and 16 years after developing nodules without evolving into rheumatoid disease.

It is subject to debate whether age of onset of these nodules favors regression (as in most cases of presentation in children), static pathology, or the progression to systemic disease. Olivé et al. present a patient with unchanged course of benign nodules for 50 years who then presented with seropositive symmetrical arthritis and new nodules, fulfilling the diagnosis of classic rheumatoid arthritis [6]. Clearly there are no certainties, and this case illustrates the importance of biopsy in diagnosis, an awareness of the potential complications, and the need for good follow-up [7].

## 4. Management

Management presents a challenge. When rheumatoid nodules present in childhood, it is highly likely the nodule will regress by itself and no intervention is required. If surgery is performed, permanent scarring or keloid formation can occur. Fewer cases demonstrate regression if nodules are present in adulthood. Therapies suggested to be discussed with the patient include surgery, DMARDs, or topical tacrolimus. Due to the lack of consensus on any established therapy we elected to treat the patient with steroids and monitor for regression of his nodules.
